# Effect of 3D Cultivation Conditions on the Differentiation of Endodermal Cells 

**Published:** 2012

**Authors:** O. S. Petrakova, V. V. Ashapkin, E. A. Voroteliak, E. Y. Bragin, V. Y. Shtratnikova, E. S. Chernioglo, Y. V. Sukhanov, V. V. Terskikh, A. V. Vasiliev

**Affiliations:** Koltzov Institute of Developmental Biology, Russian Academy of Sciences, Vavilova Str. 26, Moscow, Russia, 119334; Lomonosov Moscow State University, Faculty of Biology, Leninskie Gory 1/12, Moscow, Russia, 119991; Belozersky Institute, Moscow State University, Leninskie Gory, 1/40, Moscow, Russia, 119991; Center of Innovation and Technology of Biologically Active Compounds and Their Applications, Russian Academy of Sciences, Gubkina Str. 3/2, Moscow, Russia, 117312

**Keywords:** 3D conditions, collagen gel, differentiation, endoderm, submandibular salivary gland cells, liver progenitor cells

## Abstract

Cellular therapy of endodermal organs is one of the most important issues in modern cellular biology and biotechnology. One of the most promising directions in this field is the study of the transdifferentiation abilities of cells within the same germ layer. A method for an*in vitro*investigation of the cell differentiation potential (the cell culture in a three-dimensional matrix) is described in this article. Cell cultures of postnatal salivary gland cells and postnatal liver progenitor cells were obtained; their comparative analysis under 2D and 3D cultivation conditions was carried out. Both cell types have high proliferative abilities and can be cultivated for more than 20 passages. Under 2D cultivation conditions, the cells remain in an undifferentiated state. Under 3D conditions, they undergo differentiation, which was confirmed by a lower cell proliferation and by an increase in the differentiation marker expression. Salivary gland cells can undergo hepatic and pancreatic differentiation under 3D cultivation conditions. Liver progenitor cells also acquire a pancreatic differentiation capability under conditions of 3D cultivation. Thus, postnatal salivary gland cells exhibit a considerable differentiation potential within the endodermal germ layer and can be used as a promising source of endodermal cells for the cellular therapy of liver pathologies. Cultivation of cells under 3D conditions is a useful model for the*in vitro*analysis of the cell differentiation potential.

## INTRODUCTION 

Investigating the plasticity of the cellular phenotype and the ability of cells to undergo transdifferentiation within a single germ layer remain a pressing issue in contemporary cell biology. Such research may be helpful not only in finding solutions to fundamental problems, such as the elucidation of the differentiation pathways in the embryogenesis process, establishment of the histogenetic relationships between different cell types, but also in outlining new approaches in regenerative medicine. 

The potential of cellular therapy for treating liver pathologies is being actively researched. Despite the relative success that has been achieved in investigations carried out on laboratory animals, no safe and sufficiently efficient approach has been found thus far [1, 2]. Today, the main task is to search for easily obtainable cells that can undergo hepatic differentiation with sufficient efficiency. In terms of experimental and clinical studies on embryonic stem cells, bone marrow- [7, 9] and adipose-derived [10–12] mesenchymal cells, as well as amniotic fluid cells [13, 14], are the ones that have been best studied [3, 6]. However, only partial transdifferentiation was demonstrated in all studies; no functionally active state has been achieved: hence, the search for an optimal source of cells to treat liver pathologies still continues. 

The salivary gland remains a relatively poorly studied source of endodermal cells. However, the availability of cellular material from the salivary gland, the possibilities of using these cells in autologic and allogenic variants, and the relatively low invasiveness of the biopsy procedure makes this source of endodermal cells promising in research. 

A sufficient amount of data on the *in vitro* cultivation of salivary gland cells of human and animal origin has been accumulated today. The *in vitro* cultivated salivary gland cells are an actively proliferating culture that can undergo a significant number of passages [15]. Salivary gland cells of human and animal origin (mouse, rat, pig) are characterized by the expression of cytokeratins 18 and 19 and, frequently, of α-fetoprotein [16, 17]. Under certain cultivation conditions, these cells acquire the ability to synthesize glucagon, albumin, or insulin [18]. 

One of the approaches used in the *in vitro* investigation of the phenotypic plasticity of cells, cell cultivation in a 3D matrix (under 3D conditions) using a type I collagen gel, is discussed in the present work. Cell cultivation in the collagen gel is used to investigate the morphogenetic potential of cells [19, 20], cellular migration [21], and to assess the cell differential potential [22]. Moreover, cultivation of pancreatic β cells in a type I 4% collagen gel contributes to higher rates of survival for these cells and increases their functional activity [23]. As for salivary gland cells, mouse postnatal salivary gland cells (PSGCs) cultured under 3D conditions (matrigel) acquire the ability to express α-fetoprotein and albumin, which is typical of hepatic differentiation [16]. Type I collagen gel, along with fibronectin, is known to be the main component of the hepatic extracellular matrix. Thus, the present study allows not only to shed some light on the differentiation potential of endodermal cells, but also assist in assessing the abilities of a collagen matrix to initiate and maintain *in vitro* hepatic differentiation of cells. 

The present study was aimed at investigating the capacity of mouse salivary gland cells to undergo hepatic differentiation during cultivation in a collagen gel. 

The comparative analysis of the properties of mouse submandibular salivary gland cells and progenitor cells isolated from the liver was carried out. The morphological, immunophenotypic, and biochemical characteristics of the cell cultures were compared under 2D and 3D cultivation conditions; the gene expression profile of these cells was also analyzed by PCR. 

## EXPERIMENTAL 

**Animals **

8–20-week-old male *C57BL* / *6 * mice were used in the present work. The animals were kept under standard conditions and had food and water available ad libitum. All the procedures were carried out in accordance with the rules established by the bioethics committee at the Koltzov Institute of Developmental Biology, Russian Academy of Sciences. 

**Isolation and cultivation of mouse postnatal submandibular salivary gland cells and mouse liver progenitor cells **

To obtain the mouse liver and submandibular salivary gland cell cultures, the animals were anesthetized by inhaled chloroform vapor and dissected. The neck and belly regions were cleaned with ethanol, skin was cut using sterile scissors, the liver and both submandibular salivary glands were extracted using tweezers. The organs were transferred into sterile test tubes containing DMEM/F12 1 :1 medium (Gibco) and 40 µg/ml of gentamicin. After the organs were minced and blood vessels and mesenchymal tissues were removed, the salivary gland and liver homogenate was washed twice with PBS, followed by treatment with a type IV collagenase solution (4 mg/ml, Sigma) in DMEM/F12 1 :1 medium for 30–40 minutes at 37°С. Cell suspensions were pipetted and passed through a 40-µm-pore-size filter to separate small cells from larger polyploid ones. The cells were washed twice with the cultivation medium. “Soft” centrifugation at 100 *g* was carried out for 2 min. The removal of erythrocytes and precipitation of primarily small cells with a higher specific density occurred under these conditions. After the supernatant was removed, the cells were re-suspended in a complete growth medium containing DMEM/F12 1:1, a 10% embryonic bovine serum (HyClone), 2 mM glutamine (Gibco), 1× ITS (Invitrogen), and 10 ng/ml EGF (Invitrogen). The cells were plated into culture dishes (Corning) coated with type I collagen at a density of 5 × 10 ^3^ cells/cm ^2^ and cultivated under standard conditions at 37°С and 5% СО _2_ . The medium was replaced daily during the first 5 days and subsequently replaced every 3 days. During the splitting procedure cells were washed twice with PBS and incubated in the presence of 0.25% trypsin for 5 min at 37 ^о^ С. The cells were split at a ratio of 1:3 and plated into type I collagen coated culture dishes. 

**Preparation of collagen gel, cultivation of cells under 3D conditions, collagen gel contraction **

Collagen gel was prepared using the conventional procedure: type I collagen was extracted from rat tails as described previously [20] and dissolved in sterile 0.1% acetic acid (5 mg/ml). First-passage cells were collected using trypsin and diluted with PBS, with allowance for the fact that the final concentration of cells be 1 × 10 ^6 ^ cells/ml of gel. All materials were cooled to +4°С prior to gel preparation; the subsequent operations were carried out under cold conditions. Sterile components were added into a separate test tube in the following sequence: 0.34 М NaOH (Sigma) until final concentration of 0.023 mM, 7.5% Na _2_ CO _3_ (PanEco) until final concentration of 0.26%, 10× DMEM (Sigma) until final concentrations of 1×, 100× glutamine (Gibco) until final concentration of 2 mM, 100× HEPES (Gibco) until final concentration of 1×, embryonic bovine serum (HyClone) until final concentration of 10%, followed by the addition of the collagen solution in acetic acid until a final concentration of collagen of 4%. The components were subsequently mixed 2–3 times, cells in PBS were added, the resulting mixture was additionally stirred once or twice, and the gel was poured into 35 mm Petri dishes (2 ml per plate). The gel was incubated in a CO _2_ incubator at 37°С for 30 min until complete polymerization. After the gel had polymerized, 2 ml of complete growth medium was added to each dish. The gel was separated from the dish walls using a pipette tip. The gel was then placed into a CO _2_ incubator; this very instant was assumed to be the zero hour of gel preparation. The cells were subsequently cultivated in the gel in the CO _2_ incubator under the standard conditions; the medium was replaced every 2 days. In order to determine the extent of gel contraction, its diameter was measured every 24 h counting from the zero hour of gel preparation. Gel containing no cells was used as the negative control of contraction. 

**Immunohistochemistry **

**Table 1:** Antibodies used in the present work

Antibody	Antigen	Company, catalog number
Primary antibodies		
CK19	Cytokeratin 19	AbCam, # ab15463-1
ALB	Albumin	R & D, # MAB1455
CYP P450	Cytochrome P450 1A1	Millipore, # AB1258
BrdU	Bromodeoxyuridine	AbCam, # ab8152
Secondary antibodies		
Alexa Fluor® 488 donkey anti-rabbit IgG (H + L)		Invitrogen, # A-21206
Alexa Fluor® 488 goat anti-mouse IgG (H + L)		Invitrogen, # A-11029

Collagen gel was incubated in the presence of 4% paraformaldehyde at room temperature for 30 min, followed by paraffin embedding in accordance with the standard methodology to prepare 40-µm-thick paraffin sections. The sections were stained with azure-eosin. 

**Immunocytochemistry **

Cells were plated onto the Petri dishes coated with the type I collagen gel 48 h prior to fixation in order to carry out immunocytochemical staining. Paraformaldehyde (4%) was used for fixation (10 min, room temperature). The dishes were subsequently washed with PBS containing 0.1% Triton X-100, followed by blocking using 1% bovine serum albumin in PBS for 30 min at room temperature. The incubation in the presence of the primary antibodies in PBS was carried out for 60 min at 37°С (or at +4°С overnight) using the dilution recommended by the manufacturer (typically, 1:200–1:500). The plates were washed with PBS 3 times for 10 min at 37°С, followed by incubation in the presence of the secondary antibodies in PBS (1:1000 dilution) for 40 min at 37°С. Washing with PBS was carried out 3 times for 10 min at 37°С with DAPI (Sigma) added during the last washing phase. The analysis was carried out on a fluorescent microscope. The antibodies used in the experiments are listed in *Table 1* . 

During the analysis of the cells after cultivation under 3D conditions for 10 days, the gel was washed with PBS, minced, and incubated in the presence of 0.075% type II collagenase (Sigma) for 60 min at 37°С. After the gel had been digested, the cells were washed using PBS, plated onto dishes coated with type I collagen, and cultivated under the standard conditions (CO _2 _ incubator, 48 h). The conventional staining procedure was subsequently carried out (see above).

**Determination of the proliferative activity of cells using bromodeoxyuridine **

The proliferative activity of cells during cultivation under 2D and 3D conditions was determined by their ability to incorporate bromodeoxyuridine (BrdU). Fifteen hours prior to fixation, BrdU (Sigma) was added to the cells until a final concentration of 10 µM. The cells were subsequently washed using PBS during cultivation under 2D conditions and fixed in 70% ethanol (30 min, +4°С). An equivalent volume of 4N HCl was then added, and incubation at room temperature for 30 min was carried out. The cells were washed with PBS until neutral pH values, incubated in the presence of the primary anti-BrdU antibodies in PBS (1:1000 dilution) for 60 min at 37°С, and subsequently washed 3 times for 10 min using PBS at 37°С, followed by incubation of the cells in the presence of secondary antibodies (1:1000 dilution) for 60 min at 37°С. The cells were then washed again 3 times for 10 min in PBS at 37°С; DAPI (Sigma) was added during the final washing phase. The cells were analyzed using a fluorescent microscope, 5,000 cells were counted for the statistical analysis. 

**Table 2:** Primers used in RT-PCR

Primer	Gene	Nucleotide sequence	Amplicon, bp	Melting temperature, °C
mGAPDH	Glyceraldehyde-3-phosphate	5’-AGG TCG GTG TGA ACG GAT TTG-3’ 5’-GGG GTC GTT GAT GGC AAC A-3’	95	62.6 62.6
mKRT8	Keratin 8	5’-TCC ATC AGG GTG ACT CAG AAA-3’ 5’-AAG GGG CTC AAC AGG CTC T-3’	242	60.1 60.0
mKRT14	Keratin 14	5’-GGC TGG AGC AGG AGA TCG CCA-3’ 5’-AGG ACC TGC TCG TGG GTG GAG ACCA-3’	90	61.0 62.0
mKRT19	Keratin 19	5’-GGG GGT TCA GTA CGC ATT GG-3’ 5’-GAG GAC GAG GTC ACG AAG C-3’	113	62.9 62.1
mAFP	Alpha fetoprotein	5’-CCA TCA CCT TTA CCC AGT TTG T-3’ 5’-CCC ATC GCC AGA GTT TTT CTT-3’	101	60.2 60.6
m1AAT	Alpha-1-antitrypsin	5’-CTC GTC CGC TCA CTA AAC AAG-3’ 5’-GCT GTC TGA GAG TCA AGG TCT T-3’	248	60.7 61.3
mTAT	Tyrosine aminotransferase	5’-AGC CGA ATC CGA ACA AAA CC-3’ 5’-GCC GAT AGA TGG GGC ATA GC-3’	146	60.9 61.3
mPEPCK	Phosphoenolpyruvate carboxykinase 1	5’-TGA CAG ACT CGC CCT ATG TG-3’ 5’-CCC AGT TGT TGA CCA AAG GC-3’	153	61.0 61.4
mALB	Albumin	5’-TGC TTT TTC CAG GGG TGT GTT-3’ 5’-TTA CTT CCT GCA CTA ATT TGG CA-3’	167	62.4 60.2
mCYP3A13	Cytochrome P450, family 3, subfamily a, polypeptide 13	5’-ATG AGG CAG GGA TTA GGA GAA G-3’ 5’-TGA GAG GAA CAG TGG ATC AAA GA-3’	189	60.7 60.7
mIns2	Insulin-2 preproprotein	5’-GCT TCT TCT ACA CAC CCA TGT C-3’ 5’-AGC ACT GAT CTA CAA TGC CAC-3’	147	60.6 60.1
mAmy	Amylase	5’-AAC GAA AGA GAA ATT GAA ACC-3’ 5’-GCC CCC ACT CCA CAC ATG TGG-3’	213	60.0 62.0

In order to determine the fraction of proliferating cells after 10 days of cultivation under 3D conditions, the cells in gel were incubated in the presence of 10 µM BrdU for 15 h, followed by gel washing with PBS, minced, and incubated in the presence of type II collagenase (Sigma) for 60 min at 37°С. Following the gel digestion , the cells were washed with PBS, plated onto dishes coated with type I collagen, and cultivated under the standard conditions in the CO _2_ incubator. After the cells were attached, a staining procedure identical to that carried out under 2D conditions was performed (see above). 

The gel was stained with anti-BrdU antibodies to analyze the features of cell growth and to reveal morphogenetic features under 3D conditions. After the cells were cultivated in the gel for 10 days, 10 µM BrdU was added to the medium for 15 h; the gel was subsequently fixed with 4% paraformaldehyde for 10 min at room temperature. The gel was then incubated in 70% ethanol (30 min, +4°С), an equivalent volume of 4N HCl was added, and incubation at room temperature for 15 min was performed. The gel was washed using PBS to obtain neutral pH values and incubated in the presence of the primary anti-BrdU antibodies in PBS (1:1000 dilution) for 16 h on a shaker at room temperature. The gel was subsequently washed 3 times for 10 min using PBS on a shaker at room temperature, followed by incubation of the cells in the presence of the secondary antibodies (1:1000 dilution) for 2 h on a shaker at room temperature. The cells were then washed 3 times for 10 min in PBS at 37°С, and DAPI (Sigma) was added during the last wash. The cells were analyzed on a fluorescent microscope. 

**Isolation of total RNA from cells **

Total RNA was isolated from the cells during the first passage, when cultivation occurred under 2D conditions and after 10 days of incubation in gel, when cells were cultivated under 3D conditions. The gel was digested using type II collagenase to isolate RNA from the cells cultivated under 3D conditions; the cells were precipitated using centrifugation. RNA was isolated using AllPrep DNA/RNA Mini Kit (Qiagen) in accordance with the manufacturer’s recommendations. RNA concentration was determined using a Qubit mini-fluorometer and the RNA Assay Kit (Invitrogen). Reverse transcriptase Superscript II (Invitrogen) and random primers were used for reverse transcription. 500 ng of total RNA was used for the reaction. 

**RT-PCR analysis of mouse salivary gland cells and mouse progenitor liver cells **

**Table 3:** Primers used in real-time PCR

Primer	Gene	Nucleotide sequence	Amplicon, bp	Melting temperature, °C
mGAPDH	Glyceraldehyde-3-phosphate	5’-AGG TCG GTG TGA ACG GAT TTG-3’ 5’-GGG GTC GTT GAT GGC AAC A-3’	95	62.6 62.6
mKRT19	Keratin 19	5’-GGG GGT TCA GTA CGC ATT GG-3’ 5’-GAG GAC GAG GTC ACG AAG C-3’	113	62.9 62.1
mAFP	Alpha fetoprotein	5’-CCA TCA CCT TTA CCC AGT TTG T-3’ 5’-CCC ATC GCC AGA GTT TTT CTT-3’	101	60.2 60.6
m1AAT	Alpha-1-antitrypsin	5’-CTC GTC CGC TCA CTA AAC AAG-3’ 5’-GCT GTC TGA GAG TCA AGG TCT T-3’	248	60.7 61.3
mTAT	Tyrosine aminotransferase	5’-AGC CGA ATC CGA ACA AAA CC-3’ 5’-GCC GAT AGA TGG GGC ATA GC-3’	146	60.9 61.3
mPEPCK	Phosphoenolpyruvate carboxykinase 1	5’-TGA CAG ACT CGC CCT ATG TG-3’ 5’-CCC AGT TGT TGA CCA AAG GC-3’	153	61.0 61.4

The RT-PCR was carried out using the ScreenMix kit (Eurogen) in accordance with the manufacturer’s recommendations. The reaction conditions were as follows: pre-incubation at 95 ^о^ С for 5 min to activate DNA polymerase, followed by 25–30 cycles: denaturation at 95 ^о^ С for 15 s; annealing at 57–59 ^о^ С for 15 s; and elongation at 72 ^о^ С for 1 min. *Table 2* lists the markers and the melting temperatures of the primers used in the experiment. 

**Agarose gel electrophoresis **

Electrophoresis was carried out in 1.5% agarose gel (Helicon) and a TAE buffer (PanEco) at a voltage of 80 V. DNA Ladder (Promega) was used as molecular weight markers in 1 kb and 100 bp increments. The probe volume was 6 µl per well. The gel was analyzed under UV light (360 nm) after staining with ethidium bromide (Sigma). 

**Quantitative PCR **

Quantitative real-time PCR was carried out using a real-time PCR kit and an EVA Green stain (Sintol) on a CFX96 real-time PCR instrument (BioRad). The reaction conditions were as follows: pre-incubation at 95 ^о^ С for 5 min to activate DNA polymerase, followed by 40 cycles: denaturation at 95 ^о^ С for 30 s; annealing at 57–59 ^о^ С for 30 s; and elongation at 72 ^о^ С for 45 s. The annealing temperature varied slightly for different genes, depending on the melting temperatures of the primers ( *Table 3* ). Fluorescence was determined in the Fam channel; the initial analysis of the results was carried out automatically using software supplied with the device. *GAPDH* mRNA was used as an internal standard relative to which the concentrations of the other mRNAs were determined. The cDNA samples of each investigated gene were (where possible) analyzed simultaneously and in parallel in the adjacent wells of the device under strictly identical conditions. 

**Determination of the rate of urea production by cells **

The rate of urea production by cellswas determined using the Urea Assay Kit (BioVision) in accordance with the manufacturer’s recommendations. The amount of urea was measured in the culture medium, and the old medium was replaced with a fresh one 24 h prior to sampling. The cells cultivated under 2D conditions were analyzed during the first passage; the media samples from cells cultivated under 3D conditions were collected on the 1 ^st^ , 5 ^th^ and 10 ^th^ days of incubation in gel. 

**Statistical analysis of the data **

All experiments were carried out in three repeats using cell cultures obtained from three different animals. Each procedure was performed under identical conditions in three technical repeats. The statistical analysis was performed using a Student’s t-test at a 95% confidence interval for the biological repeats and 99% confidence interval for the technical repeats. 

## RESULTS AND DISCUSSION 

**Morphological characteristics of mouse liver and mouse salivary gland cells cultivated under 2D and 3D conditions **

**Fig. 1:**
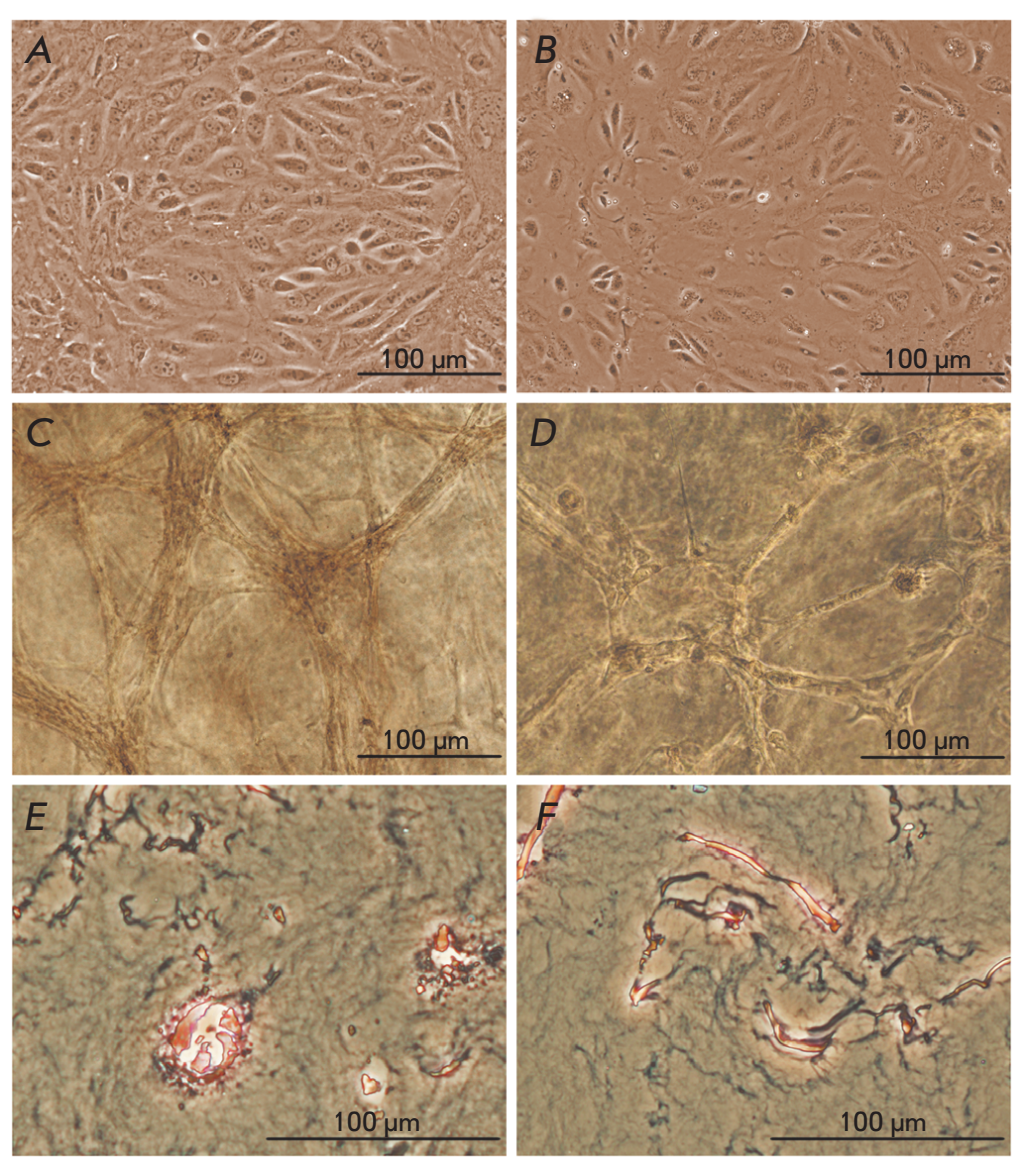
The morphology of salivary gland and liver progenitor cells under 2D and 3D cultivation conditions, phase contrast microscopy. A) PSGC, monolayer culture, 0 passage; B) PLPC, monolayer culture, 0 passage; C) PSGC, 10-th day of cultivation in 4% collagen gel, 1 passage; D) PLPC, 10th day of cultivation in 4% collagen gel, 1 passage; E) PSGC, histological section of collagen gel, 10th day of cultivation, azure-eosin staining; F) PLPC, histological section of collagen gel, 10th day of cultivation, azure-eosin staining

Following the isolation, the PLPCs were attached to the plastic coated with type I collagen for 1-2 days; the PSGCs were attached for 2-3 days. The cells were polygonal and mononuclear; they were characterized by a small size and high nucleocytoplasmic ratio. The formation of dense colonies followed. The PSGC monolayer was formed on the 5 ^th ^ day, and the PLPC monolayer was formed on the 7 ^th^ day ( *Fig. 1A,B* ). At this stage, the morphology of the cells isolated from the liver and the salivary gland was almost identical. The cell population doubling time was maximal during the 0 passage: approximately 35 h for PSGCs and 50 h for PLPCs. Following the monolayer formation and during the subsequent cultivation, the cell population doubling time was stabilized around 42 h for PSGCs and 63 h for PLPCs. Both cell cultures could undergo over 20 passages, which indicates that they have a high proliferative potential and generally consist of undifferentiated cells. 

During the first passage, PSGCs and PLPCs were incorporated into the 4% collagen gel at a concentration of 1 × 10 ^6^ cells/ml of gel. Morphological changes were observed during the subsequent 10 days of cell incubation in the gel. PSGCs became elongated and formed clusters increasing in size with the lapse of time. Bundles consisting of several dozen cells radiated from the clusters ( *Fig. 1C* ). The bundles of the PSGC cells in the gel had a tubular structure as can be seen in the paraffin sections ( *Fig. 1E* ). The PLPCs also formed clusters in the form of small bundles, although the emergence of large clusters was less pronounced ( *Fig. 1D* ). These structures are not hollow as can be seen in the sections ( *Fig. 1F* ). Some PLPCs retained their round shape, became larger, and contained many granules under 3D conditions. 

A decrease in the collagen gel size (contraction) occurs as the cellular bundles grow. It was established that the degree of contraction depends on the cytoskeleton of the cells and reflects their contractility [19]. The investigated cells acquired contractile capabilities during the differentiation in myoepithelium. Hence, the myoepithelial differentiation potential of the PSGCs and PLPCs can be determined from the degree of contraction of the collagen gel. 

**Fig. 2:**
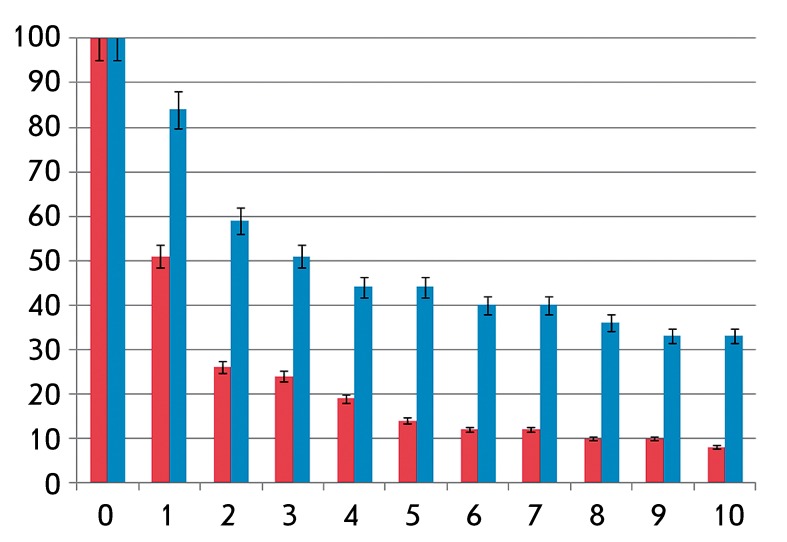
4% collagen gel contraction by salivary gland and liver progenitor cells, cell concentrations are 1 x 10 ^6^ per ml of gel, 1 passage. X-axis value is days of contraction, Y-axis value is gel area in relation to the initial gel area (%). Red columns relate to PSGC; blue columns, to PLPC

Contraction of the collagen gel was observed during the entire period of incubation of PSGCs and PLPCs under 3D conditions. PSGCs cause a significant contraction, as soon as after 5 days of cell incubation in the gel, its area decreases to 14% of the initial size ( *Fig. 2* ). Meanwhile, PLPCs contract the gel to a lesser extent: the gel area was more than 30% of its initial size on the 10 ^th^ day of incubation ( *Fig. 2* ). Therefore, the PSGCs have a higher myoepithelial differentiation potential, which is in agreement with published data [24]. 

Our data indicate that there is a similarity between the morphological characteristics of PSGCs and PLPCs during cultivation under 2D conditions. However, the morphogenetic characteristics of cells differ significantly under 3D conditions. 

**Immunocytochemical analysis of mouse liver and mouse salivary gland cells cultures under 2D conditions and after cultivation in collagen gel **

In order to determine the effect of 3D cultivation conditions on the expression of hepatic markers, immunophenotyping of PSGCs and PLPCs cultured on plastic and after incubation for 10 days in a collagen gel was carried out. Immunophenotyping of endodermal cells was performed during the first passage using the markers listed in *Table 1* .

**Fig. 3:**
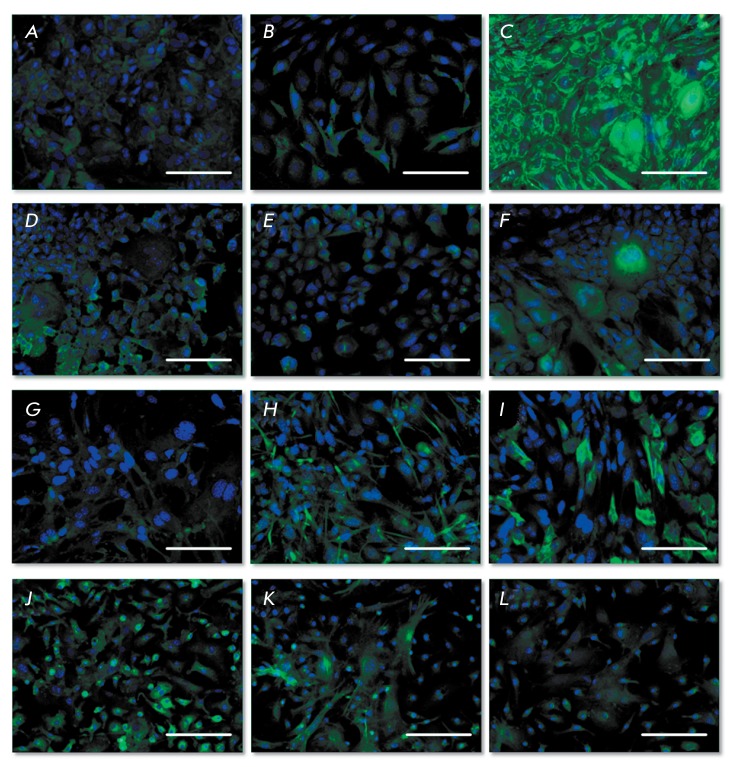
Immunocytochemistry of salivary gland and liver progenitor cells on the 1st passage, fluorescent microscopy. Cell nuclei stained by DAPI (blue color), the antigens stained by Alexa Fluor 488 (green color), bar equals to 100 microns. A) PSGC, albumin, 2D conditions; B) PSGC, cytochrome P450 1A1, 2D conditions; C) PSGC, cytokeratin 19, 2D conditions; D) PLPC, albumin, 2D conditions; E) PLPC, cytochrome P450 1A1, 2D conditions; F) PLPC, cytokeratin 19, 2D conditions; G) PSGC, albumin, after cell cultivation for 10 days in the collagen gel; H) PSGC, cytochrome P450 1A1, after cell cultivation for 10 days in the collagen gel; I) PSGC, cytokeratin 19, after cell cultivation for 10 days in the collagen gel; J) PLPC, albumin, after cell cultivation for 10 days in the collagen gel; K) PLPC, cytochrome P450 1A1, after cell cultivation for 10 days in the collagen gel; L) PLPC, cytokeratin 19, after cell cultivation for 10 days in the collagen gel

During cultivation under 2D conditions, the cells in both cultures are weakly positive with respect to albumin and hepatocyte-specific cytochrome Р450 1А1 ( *Fig. 3A, B, C, D* ). Staining for cytokeratin 19 is typical of both cultures ( *Fig. 3C,F* ); however, cytokeratin localization differs for PSGCs and PLPCs. Cytokeratin 19 localizes in the perinuclear space and under the plasma membrane in PSGCs, which can presumably be attributed to the developed system of tight junctions in these cells as they have a barrier function. In PLPCs, cytokeratin 19 localizes predominantly in the perinuclear space. Both cultures contain a small number of cells in which cytokeratin 19 is distributed throughout the entire cytoplasm. 

During the analysis of the cells cultivated for 10 days under 3D conditions, the gel was digested using type II collagenase. The cells were then plated onto dishes coated with type I collagen and cultivated under the standard conditions (CO _2_ incubator for 48 h), followed by cellular immunophenotyping. The expression level of cytochrome P450 1A1 increased due to cultivation of PSGCs in the collagen gel ( *Fig. 3H* ), whereas the expression level of albumin remained intact. The expression of cytokeratin 19 decreased, and its localization changed: in most cells, cytokeratin 19 localized in the perinuclear space ( *Fig. 3I* ). Following the incubation in the collagen gel, the expression of albumin and cytochrome P450 increased, while the expression of cytokeratin 19 decreased in PLPCs ( *Fig. 3J–L* ). 

Hence, the investigated cell cultures are characterized by a similar expression pattern of hepatic markers during cultivation under 2D conditions. The immunocytochemical analysis data indicate that hepatic markers are expressed in both cultures. However, their level of expression in the investigated endodermal cells is low; which attests to the fact that PSGCs and PLPCs are in an undifferentiated state. Both PSGCs and PLPCs express markers typical of the ductal (cytokeratin 19) and the hepatic (albumin, cytochrome P450 1A1) lineage; i.e., they have a bipotent differentiation potential, which is usually observed in oval cells (e.g., [25]). Following cultivation under 3D conditions, the expression of the hepatic differentiation markers increases, which is more evident in the case of PLPCs. The expression of ductal differentiation markers (cytokeratin 19) decreases in both cultures. 

**Determination of the proliferative capacity of mouse salivary gland and mouse liver progenitor cells under 2D and 3D conditions **

**Fig. 4:**
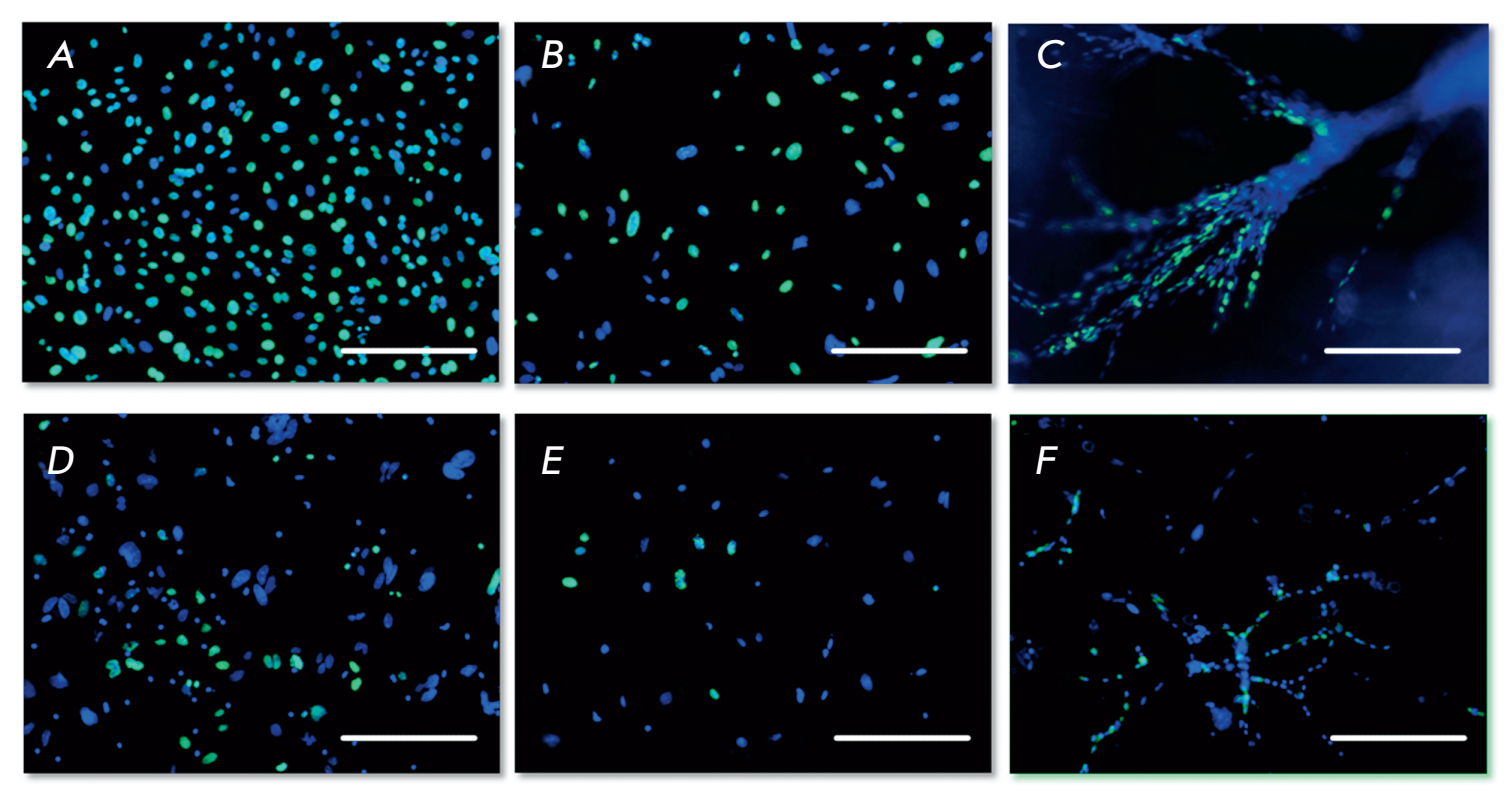
Analysis of the proliferative activity of salivary gland and liver progenitor cells under 2D and 3D cultivation conditions, 1st passage, fluorescent microscopy. Cell nuclei stained by DAPI (blue color), BrdU stained by Alexa Fluor 488 (green color), bar is equal to 100 µm. A) PSGC, 2D conditions; B) PSGC isolated from the collagen gel after cultivation for 10 days under 3D conditions; C) PSGC, 10 ^th^ day of cultivation in the collagen gel (without isolation); D) PLPC, 2D conditions; E) PLPC isolated from the collagen gel after cultivation for 10 days under 3D conditions; F) PLPC, 10 ^th^ day of cultivation in the collagen gel (without isolation)

The analysis of the proliferative capacity of cells based on the determination of BrdU incorporation demonstrated that PSGCs have a high proliferative potential. During the first passage of cultivation under 2D conditions, BrdU was incorporated into more than 90% of the salivary gland cells ( *Fig. 4A* ) and 30% of PLPCs ( *Fig. 4D* ). The PLPC population appeared to be more heterogeneous and to contain cells of variable levels of differentiation. 

On the 10 ^th^ day of cultivation in the collagen gel, BrdU was incorporated only into 52% of PSGCs and 11.5% of PLPCs ( *Fig. 4B,E* ). Hence, the cellular proliferation of both cultures under 3D conditions slows down approximately twofold. In order to determine the features of cellular growth in gel and the morphogenetic features under 3D conditions, BrdU staining was carried out without isolation of cells from the gel. As a result, no specific patterns in the distribution of proliferating cells were identified: BrdU-positive cells localized both in the outer and inner layers along the entire length of the cellular bundles ( *Fig. 4C,F* ). 

**RT-PCR for mouse salivary gland and mouse progenitor liver cells cultures under 2D and 3D cultivation conditions **

The RT-PCR analysis of the PSGC and PLPC was carried out during the first passage under 2D cultivation conditions and after cell incubation in gel under 3D conditions for 10 days. RT-PCR was performed for a wide range of markers specific to endoderm cells. The housekeeping gene *GAPDH* was used as a positive control during PCR. 

**Table 4:** RT-PCR for PSGCs and PLPCs during the 1 ^st^ passage under 2D and 3D cultivation conditions*

Primer	2D conditions		3D conditions, 10^th^day
	PSGC	PLPC	PSGC
mGAPDH	1	1	1
mKRT19	14.1	295.59	7.9
mAFP	0.01	0.12	1.19
m1AAT	0.04	0.30	3.19
mTAT	0.09	0.43	0.32
mPEPCK	0.02	0.08	0.18

* The data have been standardized with respect to GAPDH

**Fig. 5:**
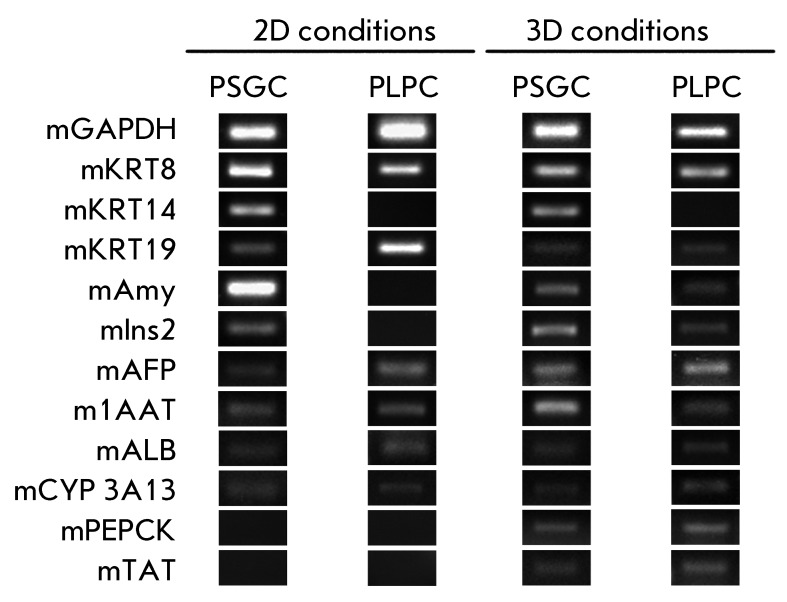
RT-PCR analysis of salivary gland and liver progenitor cells on the 1st passage under 2D and 3D cultivation conditions

According to the RT-PCR data, both PSGCs and PLPCs express cytokeratins 8, 18, 19, specific to endodermal epithelial cells during the first passage under 2D conditions. Moreover, PSGCs express cytokeratin 14, specific to the cells associated with the basement membrane, and also express amylase and insulin ( *Fig. 5* ), whereas PLPCs are negative with respect to these markers. As for the hepatocyte-specific markers, both PSGCs and PLPCs express α-fetoprotein and α-1-antitrypsin, albumin and cytochrome P450 3A13. The markers of the later stages of hepatocyte differentiation ( *PEPCK, TAT* ) were not detected under 2D cultivation conditions. 

The expression of *PEPCK* and *TAT,* which are typical of the later stages of hepatic differentiation, was detected in PSGCs and PLPCs after cultivation in the collagen gel for 10 days. Furthermore, PLPCs are characterized by expression of amylase and insulin, which are the cell markers of pancreatic differentiation. This phenotypic plasticity of liver progenitor cells is in close agreement with the published data, according to which liver and pancreatic stem cells can transdifferentiate into each other under certain *in vitro* cultivation conditions [2, 26]. 

**Quantitative Real-Time PCR for salivary gland cells cultures compared to liver progenitor cells under 2D and 3D cultivation conditions **

The comparative analysis of the PSGC and PLPC cells cultures was carried out during the first passage using quantitative real-time PCR for the markers specific to liver cells ( *Table 4* ). In addition, the expression of these genes in PSGCs was analyzed after cultivation in the collagen gel for 10 days. The data for each culture was normalized with respect to *GAPDH* , the level of expression of which was assumed to be equal to 1. 

During the first passage under 2D conditions, PSGCs express cytokeratin 19 at a relatively high level; however, its expression level in PLPCs was over 18 times higher. After the incubation in the gel, the expression of cytokeratin 19 in salivary gland cells dropped twofold. The level of expression of α-fetoprotein specific to the oval cells in both cultures was relatively low, but its expression was 10 times higher in PLPCs. The hepatic markers were expressed in the investigated cells at a relatively low level; the expression of these markers was several times higher in PLPCs. The level of α-fetoprotein expression in PSGCs increased by over 100 times after the cell incubation in gel for 10 days and significantly exceeded its expression level in PLPCs under 2D cultivation conditions. The level of expression of α-1-antitrypsin in PSGCs specific to the initial stages of hepatic differentiation increased 80 times under 3D conditions. The level of expression of the markers specific for the later stages of hepatic differentiation significantly increased as well. 

**Determination of the rate of urea production by mouse salivary gland cells and mouse progenitor liver cells under 2D and 3D cultivation conditions **

The rate of urea production by PSGCs and PLPCs under 2D and 3D cultivation conditions was analyzed to determine the functional activity of the cells under investigation. 

**Fig. 6:**
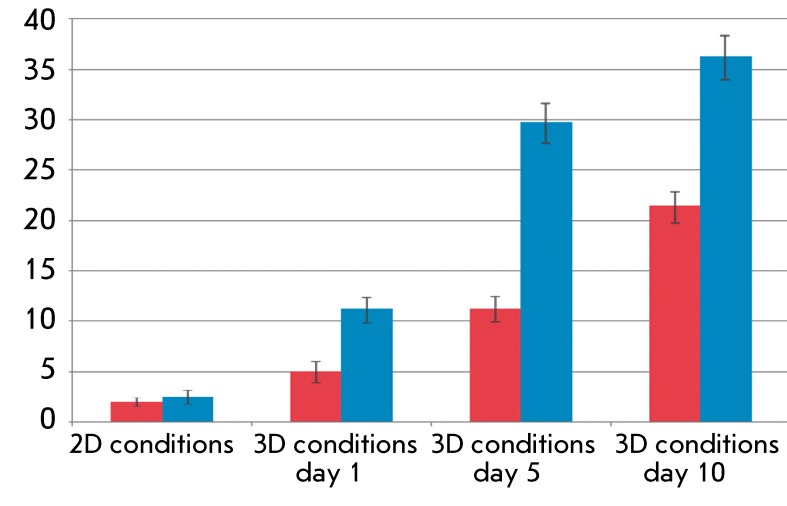
Analysis of urea synthesis by salivary gland and progenitor liver cells under 2D and 3D cultivation conditions, 1st passage. The Y-axis value is amount of urea (mM) per 1 x 10 ^6^ cells per 24 h. Red columns relate to PSGC; blue columns, to PLPC

Both cultures of endodermal cells producted virtually no urea under 2D conditions: ~2 mM of urea per 1 × 10 ^6^ of PSGCs during 24 h was detected during the first passage, and ~2.5 mM of urea was detected for PLPCs ( *Fig. 6* ). This value slightly decreases as the number of passages increases (data not shown). However, the level of urea production by both PLPCs and PSGCs gradually increases during cultivation under 3D conditions. On the 10 ^th^ day of cultivation, the rate of urea production was 15 times higher for PLPCs and 10 times higher for PSGCs as compared to the initial levels. Hence, the level of urea production by PSGCs was equal to ~60% of that by PLPCs after cultivation for 10 days under 3D conditions. 

Liver and pancreatic stem cells are known to be capable of undergoing transdifferentiation within the endodermal germ layer. Thus, the ductal cells isolated from the pancreas and transplanted into the liver differentiate into hepatocytes [27]. Oval cells can differentiate into endocrine and exocrine pancreatic cells [26]. Pancreatic islet cells can differentiate into hepatocytes in culture with increased plating density. It has been demonstrated that pancreatic acinar cells can differentiate into hepatocytes when exposed to dexamethasone [2]. However, very little data is available about the differentiation potential of salivary gland cells. A fivefold increase in albumin expression is observed in cell cultures derived from pig salivary glands after such cells are stimulated with nicotinamide [18]. Mouse salivary gland cells transplanted into the liver through the portal vein are capable of engrafting the liver and producing albumin and α-1-antitrypsin [16]. 

Generally speaking, our results have confirmed the high phenotypic flexibility of mouse salivary gland cells within the endodermal germ layer. PSGCs undergo significant and specific hepatic differentiation in the collagen gel without any additional stimulation with growth factors and cytokines. 

## CONCLUSIONS 

A mouse postnatal submandibular salivary gland cell culture was obtained and compared to mouse postnatal progenitor liver cells under 2D and 3D cultivation conditions. 

Mouse postnatal salivary gland cells and mouse progenitor liver cells are endodermal epithelial cells. PSGCs are ductal cells characterized by a high proliferative potential. The PLPC population is heterogeneous and contains cells at various differentiation stages. Both cultures possess the bipotent potential of hepatic and ductal differentiation. Moreover, PSGCs are capable of pancreatic differentiation, while PLPCs acquire this ability during cultivation in a collagen gel. 

PSGCs and PLPCs are considerably similar in terms of the expression of various cellular markers and seem to possess similar differential potentials. In general, PSGCs and PLPCs are characterized by a significant phenotypic flexibility and the ability to undergo transdifferentiation within the endodermal germ layer. 

Under 3D cultivation conditions, PSGCs and PLPCs undergo differentiation, which is characterized by a slowdown of cellular proliferation and an increase in the expression level of differentiation markers. Under 3D conditions, PSGCs are characterized by a decrease of the expression of ductal markers and an increase in the expression of hepatic markers in a similar degree with PLPCs. 

Cell culturing in a collagen gel is a convenient model of *in vitro* analysis of the cellular differential potential. During cultivation in a collagen gel, postnatal salivary gland cells undergo hepatic differentiation in the absence of any additional stimulation by cytokines and growth factors. Hence, the investigated postnatal salivary gland cells can undergo hepatic transdifferentiation and become a convenient source of cells for the cellular therapy of liver pathologies. 

## Abbreviations

2D conditions: two-dimensional conditions
3D conditions: three-dimensional conditions
BrdU - 5-bromo-2’-deoxyuridine; cDNA: complementary deoxyribonucleic acid
DAPI - 4’,6-diamidino-2-phenylindole; DNA: deoxyribonucleic acid
EGF: epidermal growth factor
ITS: insulin–transferrin–selenium
mRNA: messenger ribonucleic acid
PLPC: postnatal liver progenitor cells
PSGC: postnatal salivary gland cells
RNA: ribonucleic acid
Real-time PCR: real-time polymerase chain reaction
RT-PCR: reverse transcription polymerase chain reaction
